# Metabolic Effects of Dietary Glycerol Supplementation in Muscle and Liver of European Seabass and Rainbow Trout by ^1^H NMR Metabolomics

**DOI:** 10.3390/metabo9100202

**Published:** 2019-09-27

**Authors:** Mariana Palma, Ludgero C. Tavares, João Rito, Luís F. Henriques, João G. Silva, Rodrigo Ozório, Miguel A. Pardal, Leonardo J. Magnoni, Ivan Viegas

**Affiliations:** 1 Centre for Functional Ecology, Department of Life Sciences, University of Coimbra, 3000-456 Coimbra, Portugal; 2 Center for Neuroscience and Cell Biology, University of Coimbra, 3004-517 Coimbra, Portugal; 3 Department of Life Sciences, University of Coimbra, 3000-456 Coimbra, Portugal; 4 CIIMAR—Interdisciplinary Centre of Marine and Environmental Research—University of Porto, 4050-123 Porto, Portugal

**Keywords:** aquaculture, aquafeed, diet optimization, fish metabolism

## Abstract

The sustainable growth of fish aquaculture will require the procurement of non-marine feed sources. Glycerol is a potential feed supplement whose metabolism may spare the catabolism of dietary amino acids, thereby extending the use of the feed protein to other physiological functions such as growth. In the present study, the effects of dietary glycerol supplementation on the muscle and liver metabolomes of rainbow trout (*Oncorhynchus mykiss*) and European seabass (*Dicentrarchus labrax*) were evaluated. Fish juveniles were fed diets with 0%, 2.5%, and 5% glycerol. Muscle and liver aqueous fractions were extracted and ^1^H NMR spectra were acquired. Metabolite profiles derived from the ^1^H NMR signals were assessed using univariate and multivariate statistical analyses. The adenylate energy charge was determined in the muscle. For both species, the muscle metabolite profile showed more variability compared to that of the liver and was most perturbed by the 5.0% glycerol diet. For the liver metabolite profile, rainbow trout showed fewer differences compared to European seabass. No differences were observed in energy charge between experimental groups for either species. Thus, rainbow trout appeared to be less susceptible to tissue metabolite perturbations, compared to seabass, when the diet was supplemented with up to 5% glycerol.

## 1. Introduction

Aquaculture is an important source of animal protein for human consumption. It is recognized as a way to generate food products with high safety and nutritional levels, while ensuring sustainable economic, social, and environmental development [1]. However, aquaculture is still dependent on ingredients from wild sources, especially for carnivorous fish that have high protein requirements [2]. Improving the sustainability of aquaculture will increasingly rely on the substitution of marine-derived ingredients with alternative components. On the one hand, these alternative substrates should be readily available, easily incorporated into aquafeed, and highly digestible. However, on the other hand, aquafeeds that incorporate these new substrates need to fulfil fish nutritional and metabolic requirements to ensure optimal growth rates, flesh quality, and welfare. Since these may vary between different species of carnivorous fish, species-specific formulations need to be developed [3].

As a novel aquafeed substrate, glycerol has been proposed to be able to compete with dietary amino acids regarding the production of hepatic glucose and glycogen via gluconeogenesis. Thus, it spares the catabolism of dietary amino acids and in principle makes them more available for other physiological functions, such as growth [4]. Glycerol has been widely tested as a feed supplement in several farmed animals, including catfish (*Ictalurus punctatus*) and Nile tilapia (*Oreochromis niloticus*), with favorable results [5,6,7,8,9,10]. However, carnivorous fish, such as trout and seabass, have higher dietary protein requirements, hence it is not clear whether glycerol supplementation will be as effective for these species.

Tissue metabolite profiling using nuclear magnetic resonance (NMR) is being increasingly applied in the aquaculture field, for example, to evaluate the effects of different rearing conditions [11,12] or to determine the influence of alternative dietary ingredients on the tissue metabolome [13,14]. These and other applications were recently reviewed by Roques et al. [15].

Rainbow trout (*Oncorhynchus mykiss*) and European seabass (*Dicentrarchus labrax*) are important carnivorous fish species in aquaculture [16,17], and are representative species in the European context for freshwater and saltwater aquaculture, respectively. Therefore, our goal was to evaluate the effects of dietary glycerol supplementation in the muscle and liver metabolomes of these species. The liver has a direct role in nutrient-sensing and metabolic regulation, while the muscle is the tissue of interest for aquaculture producers, as its production rate and composition can influence the general quality and value of the final product. To address this, we followed an NMR-metabolomics-based approach.

## 2. Results

Fish from both species presented good health throughout the trials and were provided with the conditions for optimal welfare during the entire study. Feed consumption, alterations to feed behavior, and mortality were recorded daily for each tank. Fish survival was 99% and 97% for rainbow trout and European seabass, respectively.

### 2.1. Rainbow Trout

**Figure 1:**
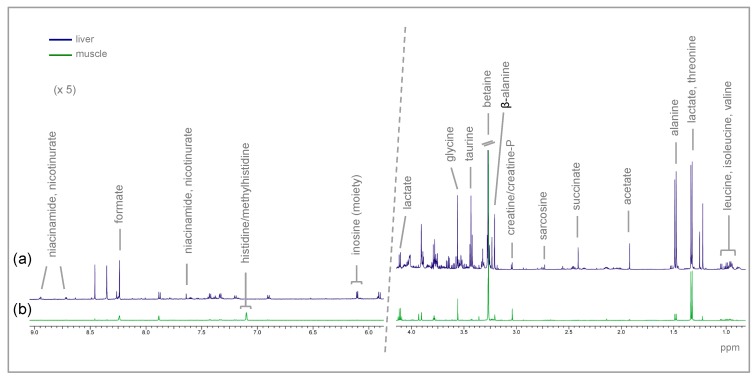
Representative NMR spectra of rainbow trout: (**a**) CPMG (Carr-Purcell-Meiboom-Gill) spectrum of the aqueous fraction of liver; and (**b**) 1D ^1^H spectrum of the aqueous fraction of muscle, with the assignments of the main identified metabolites.

Representative spectra of the aqueous fractions of the muscle and liver of rainbow trout are shown in Figure 1. Assignments of the metabolites identified in both tissues are provided as supplementary information (Table S1). Metabolites present in the highest concentrations in the muscle were betaine, glycine, and creatine/creatine-P, while in the liver, the most abundant were sarcosine, taurine, and alanine. Due to a presumptive technical error during grinding or tissue extraction, out of the 72 samples taken, two muscle samples from the T0 group (fed D0: With no glycerol), two muscle samples from the T2.5 group (fed D2.5: With 2.5% glycerol), one muscle sample from the T5.0 group (fed D5.0: With 5.0% glycerol), and one liver sample from the T0 group were removed from the analysis.

**Figure 2:**
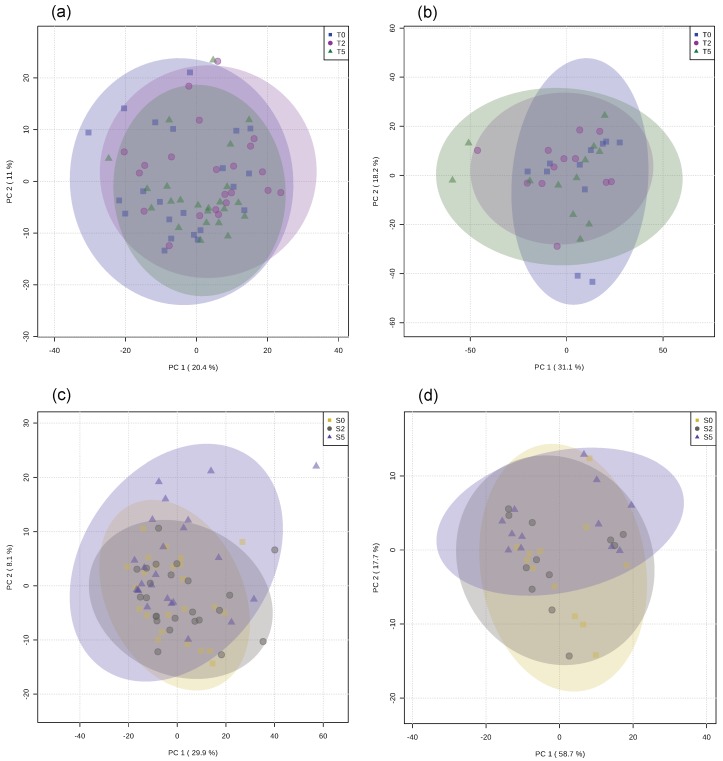
Principal component analysis (PCA) scores plot computed using multivariate analysis of: (**a**) 1D ^1^H NMR spectra of the muscle aqueous fraction, (**b**) CPMG spectra of the liver aqueous fraction of rainbow trout fed a control diet (T0: 0% glycerol) and two experimental diets (T2: 2.5% and T5: 5.0% glycerol), (**c**) 1D ^1^H NMR spectra of the muscle aqueous fraction, and (**d**) CPMG spectra of the liver aqueous fraction of European seabass when subjected to different experimental diets (S0: Control, S2: 2.5% glycerol, S5: 5.0% glycerol).

A principal component analysis (PCA) scores plot of the muscle aqueous fractions from rainbow trout is presented in Figure 2a. For this analysis, one individual from the T2.5 group was removed after being considered an outlier. Overall, the model showed no separation between the metabolite composition of samples from the three different diet conditions. The partial least squares discriminant analysis PLS model for these samples also showed no specific separation between groups and did not comply with the acceptance parameters of the validation test (supplementary information, Figure S1a).

**Figure 3:**
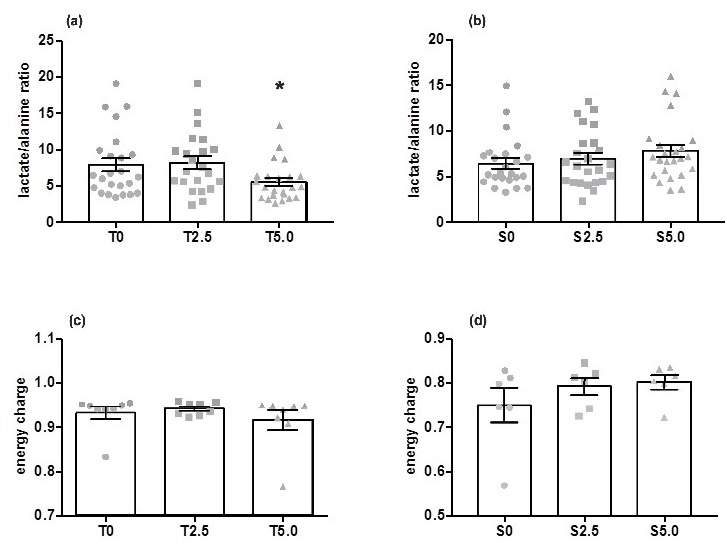
Mean values (± SEM) of the lactate/alanine ratio in the muscle aqueous fraction of (**a**) rainbow trout (T0: *n* = 22, T2.5: *n* = 22, T5.0: *n* = 23), and (**b**) European seabass (S0: *n* = 24, S2.5: *n* = 24, S5.0: *n* = 23); and energy charge in muscle of (**c**) rainbow trout (*n* = 8 per diet) and (**d**) European seabass (*n* = 6 per diet), fed three experimental diets (T0: 0% glycerol, T2: 2.5%, T5: 5.0% glycerol). Key: (*) *p* < 0.05.

Metabolites identified in the muscle and liver aqueous fraction and its fold-change variation between experimental (T2.5 and T5.0) and control diets (T0) are summarized in Table 1 and presented in detail as supplementary information (Table S2 for muscle and Table S3 for liver). Two liver samples from the T0 group were removed after being classified as outliers (GraphPad Prism 7.0a; ROUT method; Q = 1%). In muscle, both choline and betaine decreased around 0.6-fold from the T0 group to the T5.0 group. Meanwhile, niacinamide/nicotinurate increased 1.5-fold for T2.5 relative to the T0 groups. When all groups were compared together, differences were observed in choline, betaine, lactate, and niacinamide (supplementary information, Table S4). However, only choline and betaine showed differences between an experimental group, namely T5.0, and T0. The lactate/alanine ratio was calculated using the relative concentrations of these metabolites in the muscle aqueous fraction. Results showed a decrease in this ratio in the T5.0 group (Figure 3a). Concerning the adenylate energy charge, the mean values of all groups were around 0.9 and no differences were identified between them (Figure 3c).

Regarding the liver aqueous fraction, the PCA scores plot (Figure 2b) revealed no separation between the metabolome composition of the samples. The PLS model for these samples showed no evident separation and did not fulfil the parameters of the validation test (supplementary information, Figure S1b). For this analysis, one individual from the T0 group was removed after being considered an outlier. With the univariate analysis, only sarcosine revealed a slight decrease from the control group to the T2.5 group. No differences were observed when all groups were compared together (supplementary information, Table S5).

### 2.2. European Seabass

**Figure 4:**
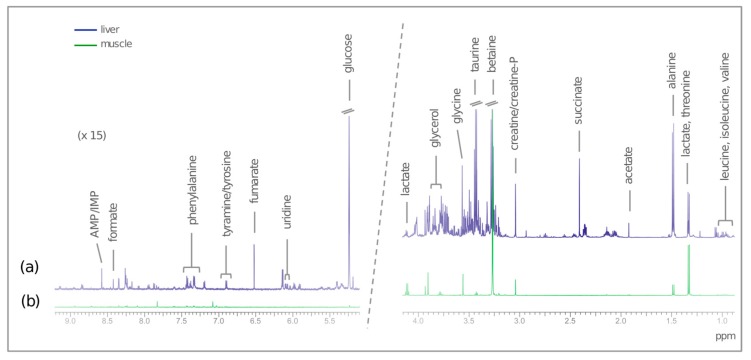
Representative NMR spectra from European seabass: (**a**) CPMG spectrum of the aqueous fraction of liver, and (**b**) 1D ^1^H spectrum of the aqueous fraction of muscle, with the assignments of the main identified metabolites.

Figure 4 shows representative spectra of the aqueous fractions of the muscle and liver of European seabass. Assignments of the metabolites identified in both tissues are presented in the supplementary information (Table S1). Metabolites present in higher concentrations in muscle were lactate, betaine, and glycine, while the higher concentrations in liver were for taurine, alanine, and glucose. Due to a presumptive technical error during grinding or tissue extraction, one muscle sample from the T5.0 group (out of a total 71 samples) was removed from the analysis.

A PCA scores plot of the muscle aqueous fractions of European seabass is presented in Figure 2c. Overall, no separation was observed between the groups. The S0 (fed D0: With no glycerol), and S2.5 (fed D2.5: With 2.5% glycerol) groups overlapped the most, while the S5.0 group (fed D5.0: With 5.0% glycerol) had a wider distribution. The PLS model for these samples showed some separation between the groups; however, the model did not meet the requirements of the validation test (supplementary information, Figure S2a).

Metabolites in the muscle aqueous fraction that showed significant differences between the different dietary glycerol levels are listed in Table 1 and are presented in detail as supplementary information (Table S6 for muscle and Table S7 for liver). There were decreases in L-leucine, isoleucine, and valine for both the S2.5 and S5.0 groups compared to the S0 group. Muscle glycerol concentrations of the S5.0 group were 11-fold higher than the S0 group. When compared together, significant differences between either S2.5 or S5.0 groups with the S0 group (supplementary information, Table S8) were observed for L-leucine, isoleucine, and valine. Glycerol showed differences between the S5.0 and S0 groups. The lactate/alanine ratio did not differ between groups (Figure 3b). Likewise, the adenylate energy charge showed no differences between groups. The mean values for the three groups were around 0.8 (Figure 3d).

Concerning the liver samples, the PCA scores plot of these samples (Figure 2d) showed no clear separation between the three groups. The PLS model also revealed a slight separation between groups but was not validated by the permutation tests (supplementary information, Figure S2b). Regarding the metabolite identification and quantification, taurine, glycine, and glycerol increased around four-, three-, and five-fold, respectively, from the S0 group to the S5.0 group. Comparison of the three experimental groups only revealed differences in glycerol between the S5.0 and S0 groups (supplementary information, Table S9).

## 3. Discussion

### 3.1. Rainbow Trout

The PCA models revealed no separation between the three groups in either the muscle or liver samples. These results indicated minor differences between the metabolome of each tissue with respect to 2.5 and 5% glycerol supplementation. Using univariate analysis, few differences were identified between controls and glycerol-supplemented groups for either tissue.

In muscle, choline and betaine decreased in the T5.0 group compared to the T0 group. Choline is an essential component of cell membranes and also prevents excessive lipid accumulation. It is also a methyl donor following oxidation to betaine, and is also a precursor of other metabolites, such as trimethylamine [18]. Therefore, decreased concentrations of choline in rainbow trout muscle could be indicative of a higher fat accumulation and poorer feed conversion to muscle protein. Glycerol was indeed described as a muscular lipogenic compound in dietary inclusion percentages of 10% and 15% in Nile tilapia [19]. A decreased choline bioavailability due to its consumption by gut microbiota was also reported for blunt snout bream (*Megalobrama amblycephala*) [20]. Our study also observed reductions in betaine levels associated with glycerol supplementations. Since betaine is produced by choline oxidation [18], this is consistent with our observed decrease of choline. In a previous study on European seabass muscle fed with a higher carbohydrate content (raw and gelatinized starch), choline and betaine also showed variations with similar trends, even if it was only significant for choline [14]. Increased values of niacinamide/nicotinurate were observed between the T2.5 and T0 groups. Due to the superimposition of their chemical shifts in the aromatic region and their low concentration, it was not possible to accurately differentiate these molecules using ^1^H NMR. Since niacinamide is the active form of the vitamin B niacin, while nicotinurate is one of its derivates, our discussion will focus on the physiological function of niacinamide. Niacinamide is a component of NAD(P), being involved in the transport of electrons in hydrogen transport complexes. Variations in niacinamide could then be related to changes in energy production mechanisms in the muscle of the T2.5 group. Regarding the energetic state of the tissues, the lactate/alanine ratio is indicative of the tissue redox state and the NADH/NAD^+^ equilibrium [21]. The slightly decreased lactate/alanine ratio for the T5.0 group could be indicative of elevated cytosolic NADH/NAD^+^, which among other things, would constrain muscle glycolytic fluxes [21]. The energy charge of muscle was around 0.9 for the three groups, reflecting a normal energetic state [22]. In general, while dietary glycerol incorporation induced some changes in energy-related muscle metabolites, it nevertheless seems to have had minor effects on the overall energetic state of the muscle.

In liver, sarcosine levels slightly decreased from the control group to the T2.5 group. Sarcosine is an intermediate in the conversion of dietary choline (via betaine) to glycine [23,24]. Although this change was observed in the liver of the T2.5 group while the variations in choline and betaine were observed in muscle of the T5.0 group, it seems that in general, the levels of glycerol supplementation used in our study had measurable effects on choline metabolism in both muscle and liver.

### 3.2. European Seabass

In European seabass, both PCA models revealed no separation between experimental groups in either the muscle or liver. In the muscle, the S0 and S2.5 groups showed an almost complete overlap, while the S5.0 group had a wider distribution. For the liver, all groups presented a similar spread distribution. Thus, our results indicate minimal effects of glycerol supplementation up to 5.0% on the metabolome compositions of liver and muscle.

Univariate analysis in muscle metabolite concentrations revealed a decrease in leucine, isoleucine, and valine in both the S2.5 and S5.0 groups when compared with the control group. The three metabolites varied in the same range in both groups. Leucine, isoleucine, and valine are all branched amino acids and three of the ten identified as indispensable for growth and for optimal feed efficiency in several fish species [25]. These amino acids can be catabolized by the Krebs cycle and gluconeogenesis (via acetyl-CoA and pyruvate) when the amount of dietary carbohydrates is reduced [20]. The decreased concentrations of these amino acids in the muscle of the S2.5 and S5 groups could indicate an increased rate of disappearance via catabolism.

In seabass, glycerol concentrations were significantly increased in both the muscle and liver of the S5.0 group, which is indicative of glycerol incorporation from the diet. Glycerol incorporation and turnover in fish has already been described in European seabass, rainbow trout, and Nile tilapia, and results are indicative of its hepatic conversion to glucose through gluconeogenesis [4,26] and the Krebs cycle [27], and as an efficient way to replenish hepatic glycogen in fasted European seabass [4]. As further discussed, results on liver metabolomics could be suggestive of the metabolic use of glycerol as an alternative substrate for energy production. Regardless of these changes in muscular metabolites, glycerol supplementation did not appear to perturb the general energetic state of the tissue, as seen by a normal energy charge (≈0.8) for all groups [22].

As mentioned before, increased values of taurine and glycine in the S5.0 group’s livers could indicate changes in anabolic-related processes. Taurine has diverse physiological functions, including osmoregulation, membrane stabilization, and regulation of the bile acid composition and antioxidant activity. Glycine is a non-essential amino acid and an important osmolyte in fish. It is an intermediate in gluconeogenesis, fat digestion, and sulfur-amino acids metabolism [28]. Previous studies in rainbow trout referred to glycine as an activator of thyroid functions after food (protein) intake, improving nutrient absorption efficiency and promoting anabolism, though with no significant increase in growth indices [29]. Similar results were observed for both glycine and taurine in juvenile European seabass muscle when fed with two carbohydrate-enriched diets (gelatinized and raw starch) [14], though no differences were observed in the daily growth index for the digestible starch diet [30]. In the liver, significant differences were only observed in the S5.0 group, suggesting that glycerol only perturbed the metabolome at the highest concentration. Tissue-specific responses have already been observed in European seabass in the regulation of lipid-related genes in response to fasting and re-feeding [31].

### 3.3. Rainbow Trout versus European Seabass

The general muscle and liver metabolomes were similar in both species. In muscle, betaine and glycine were the dominant metabolites in both species. Betaine is a major constituent of marine organisms [32] whose major physiological functions have already been mentioned. Glycine is commonly provided through the diet and can be oxidized for energy or converted to serine. Taurine is the most abundant free amino acid in animal tissues, and comprises up to 25% of the total free amino acids in liver [33]. As part of the glucose–alanine cycle, alanine is transported from peripheral tissues, such as muscle and the intestine, to the liver at a high rate of flux, which explains its abundance in the liver of both species.

Generally, dietary glycerol inclusion seemed to promote different metabolic responses in rainbow trout and European seabass. For both species, the muscle metabolome seemed to be more sensitive to glycerol compared to that of the liver. For rainbow trout, this was only observed with the highest glycerol level, while the seabass metabolomes responded to both glycerol concentrations. Concerning the tissue energy charge, although it was not modified by glycerol for either rainbow trout or seabass, the mean value for all seabass (fed or not fed with glycerol) was significantly lower that of all rainbow trout. This suggests basal differences in energy requirements or production pathways between these species.

Regarding liver, rainbow trout had only one metabolite that was significantly altered by glycerol feeding (2.5% glycerol), while in seabass, three metabolites showed significant variations, all with 5% glycerol feeding. The trout liver metabolome seemed to be slightly influenced by lower concentrations of dietary glycerol, whereas seabass liver was more sensitive to the higher concentration.

In trout, regardless of the tissue and the dietary glycerol concentration, results were indicative of variations in choline-related metabolism. Besides, seabass seemed to have different sensitivities to the percentage of dietary glycerol depending on the body compartment. The liver metabolome was more influenced by higher dietary glycerol concentrations, while the muscle metabolome was sensitive to both concentrations. However, all alterations in seabass were in general related to protein biosynthesis pathways. Complementary data from lipid composition on both species could provide additional information on the overall effects of glycerol supplementation and provide a more complete metabolic assessment.

Only seabass that were fed with the glycerol-supplemented diets showed elevated glycerol concentrations in liver and muscle. This suggests that under these conditions, the metabolic clearance of glycerol was not matched to its rate of appearance in these tissues. It is noteworthy that the triacylglycerol turnover rate in rainbow trout is higher than for other fish species [26]. This data could help explain why dietary glycerol seemed to be more rapidly cleared by trout compared to seabass. Since the liver is one of the central organs in metabolic regulation with an influence on the whole-body steadiness, rainbow trout seemed to be less susceptible to hepatic alteration, and consequently, this should also be reflected in other tissues.

### 3.4. Relevance in the Aquaculture Context

Different perturbations of liver and muscle metabolomes by the dietary incorporation of glycerol were observed for two carnivorous fish species: The rainbow trout and European seabass. These observations reinforced the concept that aquafeed optimization may require a species-specific approach, particularly for new aquafeed ingredients. For this work, we chose two carnivorous species whose tolerances to and requirements for dietary carbohydrates are very similar [34]. Rainbow trout juveniles have a slightly lower protein requirement when compared with European seabass juveniles, which could explain their different responses to dietary glycerol. Temperature and salinity also influence metabolic rates, and should also be taken into account in response to dietary changes.

Regarding the effects on the general metabolism, rainbow trout seemed to adapt better than seabass to the dietary glycerol supplementations of 2.5% and 5.0%. Data from growth rate, microbiome profile, and feed digestibility will also be important to fully understand how these changes affect overall fish growth and wellbeing for a given species. Concerning the organoleptic qualities of the fillet, it is not clear whether changes in water-soluble metabolites result in noticeable changes to taste and texture. A previous study on gilthead seabream (*Sparus aurata*) fed 5% dietary glycerol found no significant effects on aroma, color, and texture of the muscle [35].

Glycerol could be used as a supplement for finishing or growth diets for cultured fish. Muscle lipidomic information will be important for a more complete assessment of this dietary modification as fat content can change the organoleptic qualities of the fillet and the consumer acceptance of the product.

Our present results provide novel information on the metabolic effects of dietary glycerol inclusion in liver and muscle of rainbow trout and European seabass, which can not only be used to complement data from other -omics approaches, but also be applied to guide diet optimization and new aquafeed development.

The NMR-metabolomics approach generates a standardized data output, is based on simple extraction procedures, and can be performed with short NMR acquisition times. For aquaculture research, this approach provides a highly practical method for assessing tissue metabolite composition in any fish species.

## 4. Materials and Methods

### 4.1. Diet Formulation

Three isoproteic, isolipid, and isoenergetic diets were formulated while fulfilling the known nutritional requirements of both species (Sparos Lda., Loulé, Portugal). Briefly, a control diet with no glycerol (D0), and two experimental diets, supplemented with 2.5% (D2.5) or 5.0% (D5.0) glycerol, were used. Further details can be found as supplementary information (Table 2). For the sake of context, D0, D2.5, and D5.0 will hereafter be referred to as T0, T2.5, and T5.0 or S0, S2.5, and S5.0, when addressing the rainbow trout or European seabass experiments, respectively.

### 4.2. Fish Handling and Sampling: Rainbow Trout

Rainbow trout (*Oncorhynchus mykiss*) experiments were carried out at the Experimental Research Station of the University of Trás-os-Montes e Alto Douro (UTAD, Vila Real, Portugal). Nine groups of 25 fish (average initial body weight: 20.2 ± 0.1 g) were distributed in a non-predetermined pattern between nine fiberglass tanks (volume: 300 L, water flow rate: 120 L h^−1^), in a flow-through freshwater system (15 ± 1 °C temperature, 6.7 ± 0.1 pH, 8.6 ± 0.7 mg L^−1^ dissolved O_2_, exposed to a natural photoperiod). The diets (T0, T2.5, and T5.0) were haphazardly assigned to three tanks each and fish were hand-fed twice daily until apparent satiety for 8 weeks. After concluding samplings for other experimental procedures, 72 fish (24 per diet, 8 from each replicate tank) were transferred into tanks with fresh water enriched with ≈4% ^2^H_2_O in a well-aerated recirculation system equipped with an external filtering unit and UV unit for the last 6 days of feeding. Twenty-four hours after the last meal, animals were anesthetized in tank water containing 0.1 g L^−1^ of MS-222 (Sigma-Aldrich, Stainheim, Germany) and enriched with ≈4% ^2^H_2_O. Fish were measured, weighed, and liver was extracted and freeze-clamped in liquid nitrogen in pools of two livers per sample. The average final body weights were: Group T0 = 87.5 ± 2.1 g, group T2.5 = 85.3 ± 2.9 g, and group T5.0 = 79.4 ± 1.4 g. After sacrifice using a cervical section, muscle from the epaxial quadrant was collected, weighed, and freeze-clamped in liquid nitrogen. Samples were stored at −80 °C until further processing.

### 4.3. Fish Handling and Sampling: European Seabass

European seabass (*Dicentrarchus labrax*) experiments were carried out at the Interdisciplinary Centre of Marine and Environmental Research (CIIMAR, Porto, Portugal). Fifteen groups of six to eight fish (average body weight = 51.4 ± 1.5 g) were distributed in a non-predetermined pattern between 15 glass tanks (volume 80 L), in a recirculated saltwater system (30‰ salinity, 22 ± 1 °C temperature, 8.0 ± 0.1 pH, 7.9 ± 0.2 mg L^−1^ dissolved O_2_, exposed to a 12-h light/12-h dark photoperiod). The diets (S0, S2.5, and S5.0) were haphazardly assigned to five tanks each and fish were hand-fed twice daily until apparent satiety for 6 weeks. After concluding samplings for other experimental procedures, 72 fish (24 per diet, 4–5 from each replicate tank) were transferred into tanks with saltwater enriched with ≈4% ^2^H_2_O in a well-aerated recirculation system equipped with an external filtering unit and UV unit for the last 6 days of feeding. Twenty-four hours after the last meal, fish were sampled as described above for rainbow trout. The average final body weights were: Group S0 = 87.3 ± 7.3 g, group S2.5 = 75.4 ± 4.3 g, and group S5.0 = 87.6 ± 7.3 g.

### 4.4. Tissue Metabolite Extraction

Muscle and liver samples (*n* = 24 and *n* = 12, per diet each species, respectively) were powdered with a mortar and pestle chilled with liquid nitrogen, avoiding thawing during the entire process. Tissue aqueous fraction extraction was performed in all samples following the MTBE (Tert-Butyl Methyl Ether) extraction method, as described previously [36], transferred to a new vial, and lyophilized. Dried samples were kept at room temperature until further analysis.

### 4.5. NMR Acquisition

The aqueous fraction samples were re-suspended in 200 μL of 99.8% ^2^H_2_O and 40 μL phosphate buffer (50 mM, pD 7.41, with 4.966 mM 3-(Trimethylsilyl) propionic-2,2,3,3-d_4_ acid sodium salt (TSP), with sodium azide, in D_2_O). Samples were transferred into 3 mm NMR tubes. Proton (^1^H) NMR spectroscopy was conducted on a Varian VNMRS 600 MHz (Agilent, Santa Clara, CA, USA) spectrometer equipped with a 3-mm ID-PFG broadband probe at 298 K. For muscle samples, spectra were collected using a ^1^H-Presat pulse sequence (spectral width: 7200 Hz, relaxation delay: 4 s, saturation time: 3 s, acquisition time: 3 s). For liver samples, spectra were collected using a Carr-Purcell-Meiboom-Gill (CPMG) pulse sequence (spectral width: 7200 Hz, relaxation delay: 2 s, saturation time: 2 s, acquisition time: 3 s, 256 ecopulses, 512 ms ecotime). Additional J-resolved spectra were acquired in selected samples, to assist with assignment. All spectra were processed in the ACD/NMR Processor Academic Edition from ACD\Labs 12.0 software (Advanced Chemistry Development, Inc., Toronto, ON, Canada) applying: Zero-filling to 65 k, line broadening of 0.2 Hz, and phasing and baseline correction. The chemical shifts were referenced to the TSP peak at 0 ppm.

### 4.6. Spectra Analysis

#### 4.6.1. Untargeted Analysis

Spectral binning was performed in ACD/NMR Processor Academic Edition from ACD\Labs 12.0 software (Advanced Chemistry Development, Inc.) using uniform binning with a 0.04 ppm width from −0.5 to 10 ppm. Regions for water (4.69–5.02 ppm in rainbow trout, 4.66–4.88 ppm in seabass) and TSP (−0.29 to 0.04 ppm in rainbow trout; −0.29 to 0.53 ppm in seabass) were excluded. Multivariate analysis was performed using MetaboAnalyst 4.0 software (https://www.metaboanalyst.ca) [37] for principal component analysis (PCA) and partial least squares discriminant analysis (PLS). For the PLS analysis, Q^2^ (predictive ability of the model), R^2^ (goodness of the fit), and the *p*-value of the permutation test (1000 permutations) were considered as the quality parameters of each model. Models were accepted as valid for Q^2^ above 0.5 and *p*-value < 0.05 [38]. All ellipses in the scores plots, for both PCA and PLS models, were drawn at the 95% confidence level.

#### 4.6.2. Targeted Analysis

Metabolite identification and quantification was performed using ACD/NMR Processor Academic Edition from ACD\Labs 12.0 software (Advanced Chemistry Development, Inc., Toronto, ON, Canada), assisted by the Chenomx NMR Suite Library version 10 (Chenomx Inc., Edmonton, AB, Canada), the Bayesil software (http://bayesil.ca), and published literature [39]. Univariate analysis was applied to the final concentration values for each metabolite in each sample/pool, for both tissues, using GraphPad Prism 7.0a software (GraphPad Software, Inc., San Diego, CA, USA). Depending on the results of the normality assumptions using the D’Agostino and Pearson normality test, ANOVA or the Kruskal–Wallis test was applied to test differences between the three groups. The fold-change (FC) values of metabolite concentrations (FC = metabolite concentration in experimental group/metabolite concentration in control group) were calculated. Depending on the results of the normality assumptions using the D’Agostino and Pearson normality test, the *t*-test (two tails, type 3) or Mann-Whitney test was applied to determine the *p*-value of each FC value. Metabolites were identified using the Metabolomics Standards Initiative (MSI) level 2 according to the guidelines for metabolite identification [40].

The metabolomics data generated during the current study were submitted to the EMBL-EBI MetaboLights database with the identifier MTBLS832 (https://www.ebi.ac.uk/metabolights/ MTBLS832).

### 4.7. Measurement of Adenine Nucleotides

Muscle samples were selected without any specific order from each experiment of both rainbow trout (*n* = 8 per diet) and seabass (*n* = 6 per diet), and powdered with mortar and pestle chilled with liquid nitrogen, avoiding thawing throughout the process. Adenine nucleotides (ATP, ADP, and AMP) were determined as described previously [41,42]. The entire procedure of the acidic extraction was carried out in ice to avoid nucleotide degradation. Adenine nucleotides were then separated on a Waters HPLC apparatus consisting of a binary pump (model 1525) and a dual-λ absorbance detector (model 2487) (Waters Company, Milford, MA, USA). The detection wavelength was 254 nm using a Lichrospher column (100 RP-18, 5 μM, with a Lichrocart 4-4, from Merch (Darmstadt, Germany)). Isocratic elution was performed with 100 mM phosphate buffer (KH_2_PO_4_, pH 6.5) and 1.2% methanol, with a flow rate of 1 mL min^−1^. The analysis of each sample required around 5 min. The integral of each peak was calculated and normalized by the peak areas of the standard. Adenylate energy charge (EC) is equal to half the average number of anhydride-bound phosphate groups per adenosine moiety, given by the equation: EC = ([ATP] + 0.5 [ADP])/([ATP] + [ADP] + [AMP]), and varies between 0 and 1 [43]. Depending on the results of the normality assumptions of the D’Agostino and Pearson normality test, ANOVA or the Kruskal–Wallis test was used to test significance.

### 4.8. Animal Welfare Disclaimer

All experimental procedures were designed and conducted under the supervision of accredited experts in laboratory animal science by the Portuguese Veterinary Authority (1005/92, DGV-Portugal, following FELASA—Federation for Laboratory Animal Science Associations category C recommendations), according to the guidelines on the protection of animals used for scientific purposes from the European directive 2010/63/UE, and approved by CIIMAR—Interdisciplinary Centre of Marine and Environmental Research (Ref. ORBEA 8-2017), for *D. labrax* and *O. mykiss*. This study was performed and supervised by accredited scientists in laboratory animal science by the Portuguese Veterinary Authority (1005/92, DGAV-Portugal) following FELASA category B and C recommendations.

## Figures and Tables

**Table 1:** Summary of the metabolites with a significant fold-change variation between groups fed with the control diet (D0) and the diets supplemented with 2.5% (D2.5) or 5.0% (D5.0) glycerol. Significance was tested using a *t*-test or Mann–Whitney test according to the results of the normality assumptions of the D’Agostino and Pearson normality test. Detailed information about all metabolites identified in the muscle and liver of rainbow trout and European seabass is presented in supplementary information (Table S2, Table S3, Table S6, and Table S7). Rainbow trout: *n* = 65, European seabass: *n* = 72. Key: (-) fold change with non-significant variation; (*) *p* < 0.05; (**) *p* < 0.01; (***) *p* < 0.001.

	Muscle	D2.5/D0	D5.0/D0	Liver	D2.5/D0	D5.0/D0
**European Seabass**	L-leucine	0.78 *	0.78 *	Taurine	-	4.11 *
	Isoleucine	0.72 ***	0.73 ***	Glycine	-	3.18 *
	Valine	0.75 ***	0.75 ***	Glycerol	-	5.57 **
	Glycerol	-	11.79 **	-	-	-
**Rainbow Trout**	Choline	-	0.62 ***	Sarcosine	0.16 *	-
	Betaine	-	0.68 ***	-	-	-
	Lactate	-	-	-	-	-
	Niacinamide/Nicotinurate	1.56 *	-	-	-	-

**Table 2:** Diets ingredients list and composition as fed basis. Key: D0—control diet, D2.5—diet with 2.5% glycerol, and D5.0—diet with 5% glycerol.

Ingredients	D0 (%)	D2.5 (%)	D5.0 (%)
Fishmeal Super Prime	10.00	10.00	10.00
Fish Protein Concentrate	5.00	5.00	5.00
Squid Meal	5.00	5.00	5.00
Soy Protein Concentrate	10.00	10.00	10.00
Pea Protein Concentrate	5.00	5.00	5.00
Wheat Gluten	7.50	7.50	7.50
Corn Gluten	7.50	7.50	7.50
Soybean Meal 48	8.50	8.50	8.50
Rapeseed Meal	5.00	5.00	5.00
Gelatinized Starch	9.00	9.00	9.00
Cellulose	5.00	2.50	0.00
Fish Oil	14.00	14.00	14.00
Vit & Min Premix PV01	1.00	1.00	1.00
Lutavit C35	0.10	0.10	0.10
Lutavit E50	0.10	0.10	0.10
Soy Lecithin	2.00	2.00	2.00
Antioxidant	0.20	0.20	0.20
Sodium Propionate	0.10	0.10	0.10
Monocalcium Phosphate	1.30	1.30	1.30
Binder	2.50	2.50	2.50
L-Histidine	0.05	0.05	0.05
L-Threonine	0.15	0.15	0.15
Chromic Oxide	1.00	1.00	1.00
Glycerol	0.00	2.50	5.00
**Total**	**100.00**	**100.00**	**100.00**
-	-	-	-
**As Fed Basis**	**D0 (%)**	**D2.5 (%)**	**D5.0 (%)**
Crude Protein	44.24	44.24	44.24
Crude Fat	17.29	17.29	17.29
Fiber	1.46	1.30	1.19
Starch	11.03	11.03	11.03
Ash	6.11	6.11	6.11
Gross Energy (MJ/kg feed)	21.21	21.21	21.21

Glycerol from rapeseed, Belgosuc, Belgium.

## Supplementary Materials

The following are available online at https://www.mdpi.com/2218-1989/9/10/202/s1, Figure S1: Partial least squares analysis (PLS) scores plot of muscle and liver of rainbow trout, Figure S2: Partial least squares analysis (PLS) scores plot of muscle and liver of European seabass, Table S1: Identified metabolites in muscle and liver of rainbow trout and European seabass with assigned peaks, Table S2: Fold-change of muscle metabolites of rainbow trout, Table S3: Fold-change of liver metabolites in rainbow trout, Table S4: Results of the comparison between groups in muscle of rainbow trout, Table S5: Results of the comparison between groups in liver of rainbow trout, Table S6: Fold-change of muscle metabolites of European seabass, Table S7: Fold-change of liver metabolites of European seabass, Table S8: Results of the comparison between groups in muscle of European seabass, Table S9: Results of the comparison between groups in liver of European seabass.
