# Supplementary material for: Metabolic Effects of Dietary Glycerol Supplementation in Muscle and Liver of European Seabass and Rainbow Trout by 1H NMR Metabolomics
Source: Metabolites. 2019 Sep 27;9(10):202. doi: 10.3390/metabo9100202 (PMC6835518; doi:10.3390/metabo9100202)
Supplement: Supplementary file 1 [file metabolites-09-00202-s001.pdf]

Supplementary Information of the Journal Article submitted to *Metabolites*, entitled "Metabolic effects of dietary glycerol supplementation in muscle and liver of European seabass and rainbow trout by ^{1}H NMR metabolomics"

Authors

Mariana Palma[1*], Ludgero C. Tavares[2], João Rito[1,2], Luís F. Henriques[1], João G. Silva[3], Rodrigo Ozório[4], Miguel A. Pardal[1], Leonardo J. Magnoni[4], Ivan Viegas[1,2]

[*] Corresponding author

Mariana Palma: mpalma@uc.pt. Centre for Functional Ecology, Department of Life Sciences, University of Coimbra, Coimbra 3000-456, Portugal.

Figure S1. Partial Least Squares Analysis (PLS) scores plot computed by multivariate analysis of: (a) 1D ^{1}H NMR spectra of the muscle aqueous fraction of rainbow trout fed three diets. Validation parameters of the model: NC = 3; R^2 = 0.73; Q^2 = 0.001; *p*-value = 0.263 (1000 permutations); and (b) CPMG spectra of the liver aqueous fraction of rainbow trout fed three diets. Validation parameters of the model: NC = 2; R^2 = 0.56; Q^2 = -0.54; *p*-value = 0.491 (1000 permutations). Key: (T0) diet with 0% glycerol; (T2) diet with 2.5% glycerol; (T5) diet with 5% glycerol.

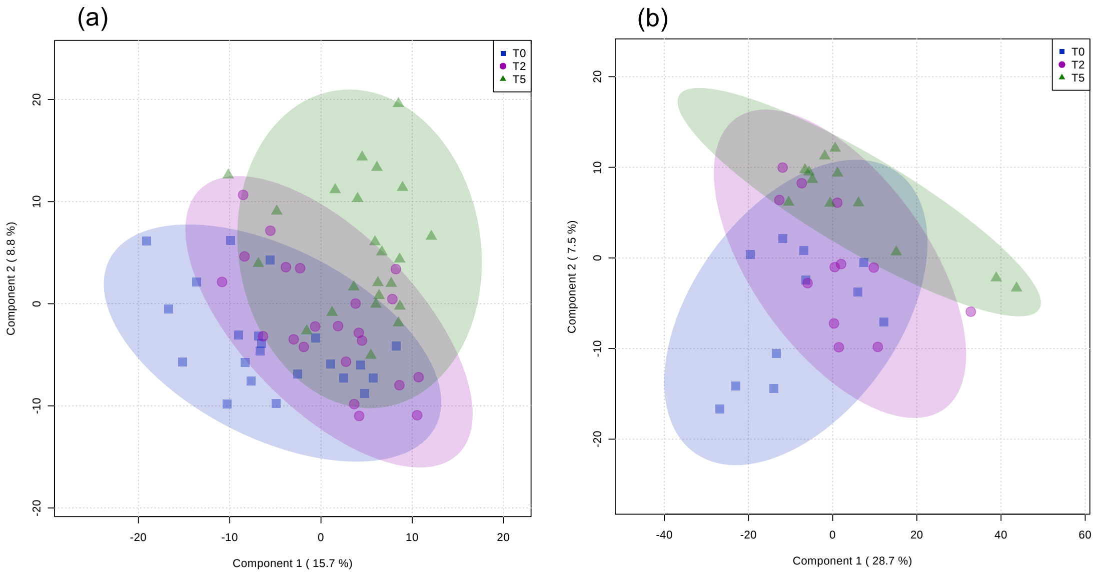

Figure S2. Partial Least Squares Analysis (PLS) scores plot computed by multivariate analysis of: (a) 1D ^{1}H NMR spectra of the muscle aqueous fraction of European seabass fed three diets. Validation parameters of the model: NC = 2; R^2 = 0.42; Q^2 = 0.04; *p*-value = 0.698 (1000 permutations); and (b) CPMG spectra of the liver aqueous fraction of European seabass fed three diets. Validation parameters of the model: NC = 5; R^2 = 0.93; Q^2 = 0.11; *p*-value = 0.051 (1000 permutations). Key: (T0) diet with 0% glycerol; (T2) diet with 2.5% glycerol; (T5) diet with 5% glycerol.

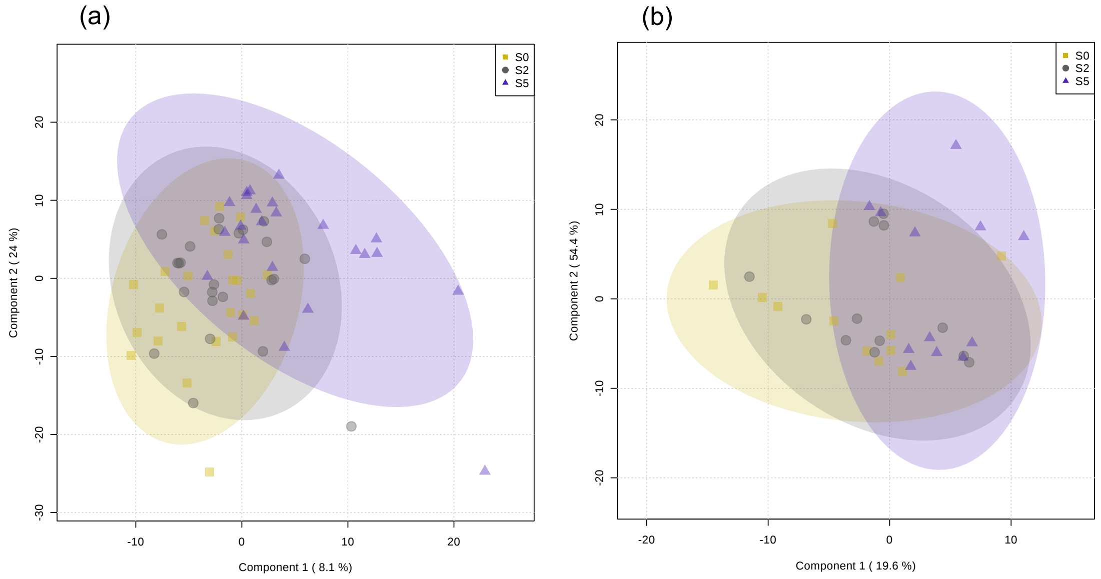

Table S1. Identified metabolites in the aqueous fraction of muscle and liver of rainbow trout and European seabass, with its assigned peaks and the compound identification code (ChEBI). Key: (-) non-identified in the tissue; (✓) identified in the tissue.

Metabolite	Assignment	δ ^{1}H ppm	Compound ID	Rainbow trout		European seabass	
		(multiplicity)	(ChEBI)	muscle	liver	muscle	liver
L-leucine	δ-CH_3	0.96 (d)	25017	✓	✓	✓	✓
	γ-CH_3	1.71 (m)					
	α-CH	3.75 (t)					
isoleucine	δ-CH_3	0.94 (t)	24898	✓	✓	✓	✓
	γ-CH_3	1.01 (d)					
	α-CH	3.67 (d)					
valine	γ-CH_3	0.99 (d)	27266	✓	-	✓	✓
	γ-CH_3	1.05 (d)					
	α-CH	3.59 (d)					
isobutyrate	CH_3	1.07 (d)	16135	–	-	✓	✓
threonine	γ-CH_3	1.32 (d)	26986	✓	-	✓	✓
	α-CH	3.59 (d)					
lactate	CH_3	1.33 (d)	78320	✓	✓	✓	✓
	α-CH	4.11 (q)					
alanine	CH_3	1.48 (d)	16449	✓	✓	✓	✓
	CH	3.78 (q)					
acetate	CH_3	1.92 (s)	15366	✓	✓	✓	✓
proline	4- CH_2	1.96-2.11 (m)	17203	–	–	✓	–
succinate	CH_2	2.42 (s)	15741	✓	✓	–	✓
glutamate	γ-CH_2	2.46 (m)	16015	–	–	–	✓
	α-CH	3.78 (q)					
β-alanine	α-CH_2	2.55 (t)	16958	–	✓	–	✓
	β-CH_2	3.18 (t)					
sarcosine	CH_3	2.73 (s)	15611	✓	✓	–	✓
asparagine	CH_2	2.86 (dd)	22653	–	–	✓	–
		2.96 (dd)					
creatine/creatine-P	CH_3	3.04 (s)	16919/17287	✓	✓	✓	✓
creatinine	CH_3	3.05 (s)	16737	✓	✓	✓	–
choline	CH_3	3.21 (s)	15354	✓	✓	✓	–
carnitine	CH_3	3.23 (s)	16347	–	–	✓	–
taurine	β-CH_2	3.26 (t)	15891	–	✓	–	✓
	α-CH_2	3.43 (t)					
betaine	CH_2	3.27 (s)	17750	✓	-	✓	✓
glycine	CH_2	3.56 (s)	15428	✓	-	✓	✓
glycerol	1'CH_2	3.66 (m)	17754	✓	✓	✓	✓
	2'CH_2						
histidine/methylhistidine	CH_3	3.82 (s)	15971/70958	✓	-	✓	-
	4'-CH	7.09 (s)					
	2'-CH	8.03 (s)					
glucose/glucose-6P	CH_2	5.24 (d)	17634/14314	–	✓	✓	✓
uridine	1'-CH	5.92 (d)	16704	–	✓	–	✓
	5'-CH	5.90 (d)					

	6-CH	7.89 (d)					
inosine (moiety)	C1H (ring)	6.11 (d)	17596	–	✓	✓	✓
fumarate	CH	6.52 (s)	18012	–	-	–	✓
tyramine/tyrosine	3',5'-CH	6.90 (d)	15760/17895	–	-	✓	✓
	2',6'-CH	7.20 (d)					
phenylalanine	2',6'-CH	7.33 (d)	28044	-	-	✓	✓
	4'-CH	7.38 (t)					
	3',5'-CH	7.43 (t)					
niacinamide/nicotinurate	C5H (ring)	7.60 (dd)	17154/7563	✓	✓	✓	✓
	C6H (ring)	8.71 (dd)					
	C2H (ring)	8.94 (d)					
formate	CH	8.46 (s)	30751	✓	✓	✓	✓
AMP/IMP	1'-CH	6.18 (d)	16027/17202	–	–	–	✓
	2-CH	8.24 (s)					
	8-CH	8.57 (s)					

Table S2. Fold change of the metabolites identified in muscle (aqueous fraction) of rainbow trout, calculated between control group (T0) and experimental groups (T2.5 and T5.0), with the correspondent p-value. T-test (1) or Mann-Whitney test (2) were applied accordingly to the normality test. Key: (ns) non-significant; (*) $p < 0.05$; (***) $p < 0.001$.

	Fold Change			
	**T2.5/T0**	**p-value**	**T5.0/T0**	**p-value**
**L-Leucine**	0.91	ns *(2)*	0.88	ns *(2)*
**Isoleucine**	0.94	ns *(2)*	0.84	ns *(2)*
**Valine**	0.93	ns *(2)*	0.89	ns *(2)*
**Alanine**	1.01	ns *(1)*	0.92	ns *(1)*
**Acetate**	0.88	ns *(1)*	0.95	ns *(1)*
**Succinate**	0.33	ns *(2)*	0.30	ns *(2)*
**Sarcosine**	0.37	ns *(2)*	0.41	ns *(2)*
**Creatine/Creatine-P**	0.86	ns *(1)*	0.92	ns *(1)*
**Creatinine**	1.02	ns *(2)*	0.82	ns *(2)*
**Choline**	0.96	ns *(2)*	0.62	*** *(2)*
**Betaine**	0.88	ns *(1)*	0.68	*** *(1)*
**Glycerol**	3.16	ns *(2)*	1.07	ns *(2)*
**Glycine**	1.06	ns *(1)*	1.06	ns *(1)*
**Threonine**	0.86	ns *(1)*	0.86	ns *(1)*
**Lactate**	1.16	ns *(2)*	0.75	ns *(2)*
**Histidine/Methylhistidine**	0.94	ns *(1)*	0.85	ns *(1)*
**Formate**	1.66	ns *(2)*	1.46	ns *(2)*
**Niacinamide/Nicotinurate**	1.56	* *(1)*	0.71	ns *(2)*

Table S3. Fold change of the identified metabolites identified in liver (aqueous fraction) of trout, calculated between control group (T0) and experimental groups (T2.5 and T5.0), with the correspondent p-value. Mann-Whitney test was applied accordingly to the normality test. Key: (ns) non-significant; (*) $p < 0.05$.

	Fold Change			
	**T2.5/T0**	**p-value**	**T5.0/T0**	**p-value**
**L-Leucine**	2.63	ns	4.75	ns
**Isoleucine**	3.01	ns	4.82	ns
**Lactate**	2.79	ns	8.84	ns
**Alanine**	4.08	ns	8.21	ns
**Acetate**	0.76	ns	5.38	ns
**Succinate**	2.08	ns	3.69	ns
**Beta-alanine**	2.57	ns	4.36	ns
**Sarcosine**	0.16	*	23.74	ns
**Creatine/Creatine-P**	1.85	ns	5.12	ns
**Creatinine**	2.10	ns	4.11	ns
**Choline**	2.56	ns	6.28	ns
**Taurine**	3.53	ns	7.41	ns
**Glycerol**	2.51	ns	8.04	ns
**Glucose/Glucose-6P**	4.02	ns	12.72	ns
**Inosine (moiety)**	1.70	ns	11.13	ns
**Niacinamide/Nicotinurate**	1.19	ns	4.73	ns
**Uridine**	1.51	ns	6.23	ns
**Formate**	2.03	ns	5.23	ns

Table S4. Metabolites identified in the muscle aqueous fraction of trout when subjected to different experimental diets. Comparison between groups was tested applying ANOVA (1) or Kruskal-Wallis test (2) according to the normality test. Tukey's or Dunn's multiple comparison tests was applied in groups with significant variations. Key: (T0) control, 0% glycerol diet; (T2.5) 2.5% glycerol diet; (T5) 5% glycerol diet; (ns) non-significant; (*) $p < 0.05$; (**) $p < 0.01$; (***) $p < 0.001$; (a, b) within a row, metabolites without a common letter differ ($p < 0.05$).

	p-value	T0	T2.5	T5.0
**L-Leucine**	ns *(2)*	-	-	-
**Isoleucine**	ns *(2)*	-	-	-
**Valine**	ns *(2)*	-	-	-
**Alanine**	ns *(1)*	-	-	-
**Acetate**	ns *(1)*	-	-	-
**Succinate**	ns *(2)*	-	-	-
**Sarcosine**	ns *(2)*	-	-	-
**Creatine/Creatine-P**	ns *(1)*	-	-	-
**Creatinine**	ns *(2)*	-	-	-
**Choline**	*** *(2)*	a	a	b
**Betaine**	** *(1)*	a	ab	b
**Glycerol**	ns *(2)*	-	-	-
**Glycine**	ns *(1)*	-	-	-
**Threonine**	ns *(1)*	-	-	-
**Lactate**	* *(2)*	ab	a	b
**Histidine/Methylhistidine**	ns *(1)*	-	-	-
**Formate**	ns *(2)*	-	-	-
**Niacinamide/Nicotinurate**	** *(2)*	ab	a	b

Table S5. Metabolites identified in the liver of aqueous fraction of trout when subjected to different experimental diets. Comparison between groups was tested applying ANOVA according to the normality test. Key: (ns) non-significant.

	p-value
**L-Leucine**	ns
**Isoleucine**	ns
**Lactate**	ns
**Alanine**	ns
**Acetate**	ns
**Succinate**	ns
**Beta-alanine**	ns
**Sarcosine**	ns
**Creatine/Creatine-P**	ns
**Creatinine**	ns
**Choline**	ns
**Taurine**	ns
**Glycerol**	ns
**Glucose/Glucose-6P**	ns
**Inosine (moiety)**	ns
**Niacinamide/Nicotinurate**	ns
**Uridine**	ns
**Formate**	ns

Table S6. Fold change of the metabolites identified in muscle aqueous fraction of European seabass, between control group (S0) and experimental groups (S2.5 and S5.0), with the correspondent p-value. T-test (1) or Mann-Whitney test (2) were applied accordingly to the normality test. Key: (ns) non-significant; (*) $p < 0.05$; (**) $p < 0.01$; (***) $p < 0.001$.

	Fold Change			
	S2.5/S0	p-value	S5.0/S0	p-value
L-Leucine	0.78	* *(1)*	0.78	** *(1)*
Isoleucine	0.72	*** *(1)*	0.73	*** *(1)*
Valine	0.75	*** *(2)*	0.75	*** *(1)*
Isobutyrate	0.79	ns *(2)*	0.91	ns *(2)*
Alanine	0.97	ns *(1)*	0.88	ns *(1)*
Acetate	1.14	ns *(1)*	1.27	ns *(1)*
Proline	1.03	ns *(2)*	1.07	ns *(2)*
Asparagine	0.68	ns *(2)*	0.89	ns *(2)*
Creatine/Creatine-P	0.96	ns *(2)*	0.86	ns *(2)*
Creatinine	0.99	ns *(1)*	1.17	ns *(1)*
Choline	0.84	ns *(2)*	0.90	ns *(2)*
Carnitine	1.26	ns *(1)*	1.18	ns *(1)*
Betaine	0.82	ns *(2)*	0.82	ns *(2)*
Glycine	0.95	ns *(2)*	0.93	ns *(2)*
Threonine	0.89	ns *(2)*	1.34	ns *(2)*
Glycerol	2.30	ns *(2)*	11.79	** *(2)*
Lactate	0.97	ns *(1)*	1.01	ns *(1)*
Glucose/Glucose 6-P	1.01	ns *(2)*	0.98	ns *(2)*
Inosine	0.92	ns *(2)*	0.90	ns *(2)*
Tyramine/Tyrosine	0.91	ns *(2)*	0.78	ns *(2)*
Histidine/Methylhistidine	0.88	ns *(1)*	1.01	ns *(1)*
Phenylalanine	0.87	ns *(2)*	0.81	ns *(2)*
Formate	1.51	ns *(2)*	1.94	ns *(2)*
Niacinamide/Nicotinurate	0.86	ns *(2)*	0.84	ns *(2)*

Table S7. Fold change of the metabolites identified in liver aqueous fraction of European seabass, between control group (S0) and experimental groups (S2.5 and S5.0), with the correspondent p-value. T-test (1) or Mann-Whitney test (2) were applied accordingly to the normality test. Key: (ns) non-significant; (*) $p < 0.05$; (**) $p < 0.01$.

	Fold Change			
	**S2.5/S0**	**p-value**	**S5.0/S0**	**p-value**
**L-Leucine**	2.01	ns *(2)*	2.71	ns *(2)*
**Isoleucine**	1.89	ns *(2)*	2.84	ns *(2)*
**Valine**	2.13	ns *(2)*	2.96	ns *(2)*
**Isobutyrate**	4.12	ns *(2)*	5.51	ns *(2)*
**Lactate**	2.15	ns *(2)*	2.54	ns *(2)*
**Threonine**	1.68	ns *(2)*	2.04	ns *(1)*
**Alanine**	1.47	ns *(2)*	2.11	ns *(2)*
**Acetate**	1.73	ns *(2)*	2.15	ns *(2)*
**Succinate**	1.66	ns *(2)*	2.37	ns *(1)*
**Glutamate**	3.04	ns *(2)*	4.82	ns *(2)*
**Beta-alanine**	3.78	ns *(2)*	3.19	ns *(2)*
**Sarcosine**	0.96	ns *(2)*	1.40	ns *(2)*
**Creatine/Creatine-P**	1.41	ns *(1)*	2.02	ns *(1)*
**Betaine**	2.24	ns *(2)*	2.87	ns *(2)*
**Taurine**	2.50	ns *(2)*	4.11	* *(1)*
**Glycine**	2.08	ns *(2)*	3.18	* *(2)*
**Glycerol**	2.07	ns *(2)*	5.57	** *(2)*
**Glucose/Glucose 6-P**	1.98	ns *(2)*	2.34	ns *(2)*
**Inosine (moiety)**	1.01	ns *(2)*	1.65	ns *(1)*
**Fumarate**	1.76	ns *(1)*	3.00	ns *(1)*
**Tyramine/Tyrosine**	1.68	ns *(2)*	2.22	ns *(1)*
**Phenylalanine**	1.66	ns *(1)*	2.14	ns *(2)*
**Uridine**	2.45	ns *(1)*	3.66	ns *(2)*
**Formate**	2.19	ns *(1)*	3.37	ns *(2)*
**AMP/IMP**	1.93	ns *(1)*	4.32	ns *(2)*
**Niacinamide/Nicotinurate**	1.72	ns *(2)*	2.71	ns *(2)*

Table S8. Metabolites identified in the muscle aqueous fraction of European seabass (*Dicentrarchus labrax*), when subjected to different experimental diets. Comparison between groups was tested applying ANOVA (1) or Kruskal-Wallis test (2) according to its conformity to normality test. Tukey's or Dunn's multiple comparison tests was applied in groups with significant variations. Key: (ns) non-significant; (**) $p < 0.01$; (***) $p < 0.001$; (a, b) within a row, metabolites without a common letter differ ($p < 0.05$).

	p-value	S0	S2.5	S5.5
L-Leucine	** *(1)*	a	b	b
Isoleucine	*** *(1)*	a	b	b
Valine	*** *(2)*	a	b	b
Isobutyrate	ns *(2)*	-	-	-
Alanine	ns *(1)*	-	-	-
Acetate	ns *(1)*	-	-	-
Proline	ns *(2)*	-	-	-
Asparagine	ns *(2)*	-	-	-
Creatine/Creatine-P	ns *(2)*	-	-	-
Creatinine	ns *(1)*	-	-	-
Choline	ns *(2)*	-	-	-
Carnitine	ns *(1)*	-	-	-
Betaine	ns *(2)*	-	-	-
Glycine	ns *(2)*	-	-	-
Threonine	ns *(2)*	-	-	-
Glycerol	** *(2)*	a	ab	b
Lactate	ns *(1)*	-	-	-
Glucose/Glucose 6-P	ns *(2)*	-	-	-
Inosine	ns *(2)*	-	-	-
Tyramine/Tyrosine	ns *(2)*	-	-	-
Histidine/Methylhistidine	ns *(1)*	-	-	-
Phenylalanine	ns *(2)*	-	-	-
Formate	ns *(2)*	-	-	-
Niacinamide/Nicotinurate	ns *(2)*	-	-	-

Table S9. Metabolites identified in the liver aqueous fraction of European seabass (*Dicentrarchus labrax*), when subjected to different experimental diets. Comparison between groups was tested applying ANOVA (1) or Kruskal-Wallis test (2) according to its conformity to normality test. Tukey's or Dunn's multiple comparison tests was applied in groups with significant variations. Key: (ns) non-significant; (*) $p < 0.05$ (a, b) within a row, metabolites without a common letter differ ($p < 0.05$).

	p-value	S0	S2.5	S5.0
L-Leucine	ns *(2)*	-	-	-
Isoleucine	ns *(2)*	-	-	-
Valine	ns *(2)*	-	-	-
Isobutyrate	ns *(2)*	-	-	-
Lactate	ns *(2)*	-	-	-
Threonine	ns *(2)*	-	-	-
Alanine	ns *(2)*	-	-	-
Acetate	ns *(2)*	-	-	-
Succinate	ns *(2)*	-	-	-
Glutamate	ns *(2)*	-	-	-
Beta-alanine	ns *(2)*	-	-	-
Sarcosine	ns *(2)*	-	-	-
Creatine/Creatine-P	ns *(1)*	-	-	-
Betaine	ns *(2)*	-	-	-
Taurine	ns *(2)*	-	-	-
Glycine	ns *(2)*	-	-	-
Glycerol	* *(2)*	a	ab	b
Glucose/Glucose 6-P	ns *(2)*	-	-	-
Inosine (moiety)	ns *(2)*	-	-	-
Fumarate	ns *(1)*	-	-	-
Tyramine/Tyrosine	ns *(2)*	-	-	-
Phenylalanine	ns *(2)*	-	-	-
Uridine	ns *(2)*	-	-	-
Formate	ns *(2)*	-	-	-
AMP/IMP	ns *(2)*	-	-	-
Niacinamide/Nicotinurate	ns *(2)*	-	-	-
